# Characterizations and morphology of sodium tungstate particles

**DOI:** 10.1098/rsos.172214

**Published:** 2018-08-08

**Authors:** F. Dkhilalli, S. Megdiche Borchani, M. Rasheed, R. Barille, S. Shihab, K. Guidara, M. Megdiche

**Affiliations:** 1 Faculty of Sciences, Laboratory of Spectroscopic Characterization and Optical Materials, University of Sfax, BP 1171, 3000 Sfax, Tunisia; 2 MOLTECH-Anjou, University of Angers/UMR CNRS 6200, 2 Boulevard Lavoisier, 49045 Angers, France; 3 Higher Institute of Computing and Multimedia of Sfax (ISIMS), Technological Center of Sfax, BP 242, SakietEzzit, 3021 Sfax, Tunisia; 4 Department of Applied Sciences, University of Technology, Baghdad, Iraq

**Keywords:** sodium tungstate, structure study, spectroscopy investigation, optical properties

## Abstract

A solid-state reaction technique was used to synthesize polycrystalline Na_2_WO_4_. Preliminary X-ray studies revealed that the compound has a cubic structure at room temperature. The formation of the compound has been confirmed by X-ray powder diffraction studies and Raman spectroscopy. Electrical and dielectric properties of the compound have been studied using complex impedance spectroscopy in the frequency range 209 Hz–1 MHz and temperature range 586–679 K. The impedance data were modellized by an equivalent circuit consisting of series of a combination of grains and grains boundary. We use complex electrical modulus M* at various temperatures to analyse dielectric data. The modulus plots are characterized by the presence of two relaxation peaks thermally activated. The morphologies and the average particle size of the resultant sodium tungstate sample were demonstrated by atomic force microscopy, scanning electron microscopy and transmission electron microscopy. The thicknesses and optical constants of the sample have been calculated using ellipsometric measurements in the range of 200–22 000 nm by means of new amorphous dispersion formula which is the objective of the present work. The results were obtained for Na_2_WO_4_ particles from experimental (EXP) and measured (FIT) data showed an excellent agreement. In addition, the energy gap of the Na_2_WO_4_ sample has been determined using ellipsometry and confirmed by spectrophotometry measurements.

## 1. Introduction

The tungsten compounds of general formula AWO_4_ and B_2_WO_4_ (A, divalent cation; B, monovalent cation) crystallize following two structural types whose nature depend on the ionic radius of the cation element (A or B). When the radius of the cation ion is greater than 1 Å, the structure crystallizes in a scheelite CaWO_4_ type. The tungsten occupies a tetrahedral site, while Ca^2+^ ions are placed at the centre of a distorted cube. By contrast, when the latter is less than 1 Å, the structure leads to a wolframite FeWO_4_ kind, that consists of oxygen octahedrons which join together by common edges made with chains [WO_4_]*_n_* and [FeO_4_]*_n_* parallel to the axis *Oz*. These chains are interconnected by common vertices [1].

Sodium tungstate (Na_2_WO_4_) and sodium molybdate (Na_2_MoO_4_) crystals are isostructural, and they belong to the class of spinel crystals with general formula Na_2_X*_n_*O_3*n*+1_ (X = W, Mo) [2,3]. These materials have attracted a great deal of interest because they exhibit a rich polymorphism [4]. Sodium tungstate Na_2_WO_4_ has been used for many applications such as the preparation of coated electrodes for electrocatalysis and as a fire retardant for fabrics [5]. In organic chemistry, sodium tungstate is used as a catalyst for epoxidation of alkenes and oxidation of alcohols into aldehydes or ketones. It is also known for its anti-diabetic effects; researchers have identified the pathways through which sodium tungstate improves pancreatic function and β cell proliferation [6].

These kinds of samples Na_2_X*_n_*O_3*n*+1_ have been studied for a long time in order to understand the physical and chemical behaviour, such as structure, phase transition and vibrational investigation [7,8]. In Na_2_WO_4_ and Na_2_MoO_4_ systems which were made by Pistorius [4], it has been reported that Na_2_WO_4_ exhibits at least one first-order phase transition at around 901 K. It was suggested that the new phase has an orthorhombic symmetry, possibly *P*_nam_. It was also suggested that another phase exists, which is stable only between 860.6 and 861.8 K [9]. In this paper, sodium tungstate particles were prepared by a solid-state reaction. We report the electrical properties and mechanism of conduction of Na_2_WO_4_ elaborated by a solid-state method at 630 K using impedance spectroscopy. In addition, the results show for the first time, to our knowledge, the optical properties of the Na_2_WO_4_ sample using spectroscopic ellipsometry (SE) in the range of 200–2200 nm with a step of 1 nm by means of the Delta Psi2 software based on a new amorphous dispersion formula at room temperature.

## 2. Experimental section

The polycrystalline sample of Na_2_WO_4_ was prepared with a high-temperature solid-state reaction technique using high-purity Na_2_CO_3_ and WO_3_ compounds in a suitable stoichiometry. The powder was thoroughly mixed in an agate mortar for 3 h and then calcined at 450°C for 6 h in a pure alumina crucible. After cooling, it was again powdered thoroughly during 2 h and then recalcined at 600°C during 6 h. Finally, the recalcined powder was used to make pellets with a diameter of 8 mm and a thickness of 1.6 mm. The pressed pellets were sintered at 650°C during 7 h. The density of the obtained sintered pellets compounds to 95% of the theoretical value. The quality (crystallinity) and formation of the compound were checked by the X-ray diffraction (XRD) technique.

XRD of the calcined powder of the compound was recorded at room temperature (27°C) with a Philips X-ray powder diffractometer (PM 9920) with CuK*_α_* (*λ* = 1.5418°A) radiation in a wide range of Bragg angles (10 ≤ 2*θ* ≤ 80) at a scanning rate of 2° min^−1^.

An infrared spectroscopic study is very important to confirm the presence of O–W–O groups and to determine their modes of vibration. The apparatus used was a Perkin–Elmer FTIR spectrometer Spectrum 100. The spectral range is between 1400 and 400 cm^−1^.

The impedance spectroscopy permits us to resolve the contributions of various processes such as bulk, grain boundary and electrode effect in the specified frequency domain. In addition, it was used to estimate the resistance and the capacitance associated with the solids.

Electrical impedances were measured as a function of both temperature and frequency. This measurement is done in the frequency ranging from 209 Hz to 1 MHz with the TEGAM 3550 and in the ranges of temperature 586–679 K.

Both the flat faces of pellet samples were electroded with high-purity fine silver particle paste for all the electrical and dielectric measurements. Two platinum electrode configurations were used to perform the electrical measurements.

The digital vernier calliper (0–150) mm range is used to measure the thickness of the Na_2_WO_4_ sample. We found a thickness of 0.15 mm close to the ellipsometry results of 0.154 mm. It can be seen that the standard deviation (*σ*) between the exact and measured values of the Na_2_WO_4_ particles is 0.0028. The lower the standard deviation, the closer the data points tend to be to the mean (or expected value).

## 3. Results and discussions

### 3.1. X-ray powder diffraction

**Figure 1.**
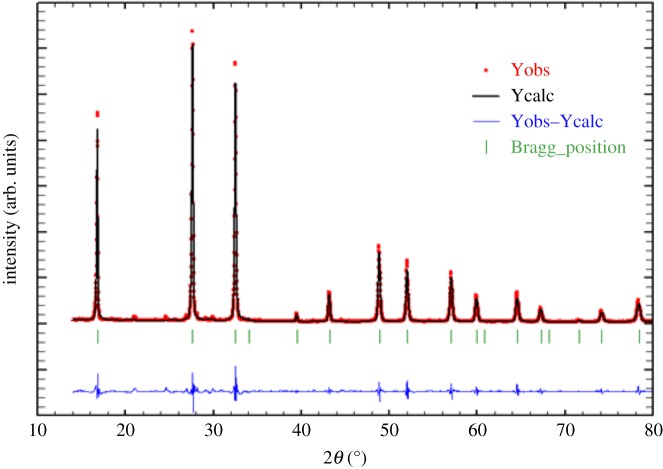
XRD of Na_2_WO_4_ in the 2*θ* range (10–80)°.

**Figure 2.**
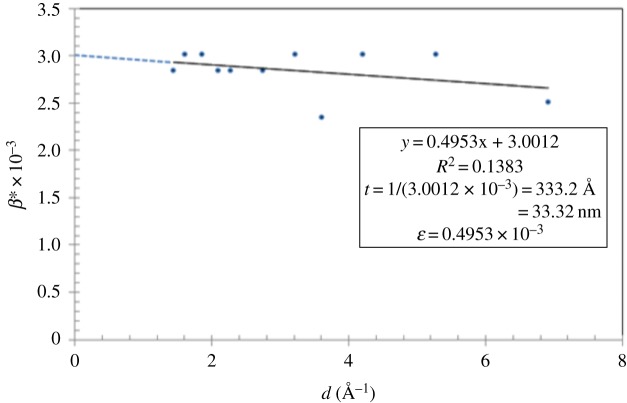
X-ray results for Na_2_WO_4_ sample.

The room temperature XRD pattern is shown in figure 1. The indexing of all peaks of the diagram was made after several optimizations using the program Fullprof with the Rietveld method. The X-ray powder diffractogram reveals that the synthesized compound crystallizes in the cubic space group Fd-3 m with $\alpha =90^{\circ },\beta =90^{\circ },\gamma =90^{\circ }$ and the unit cells: *a* = *b* = *c* = 9.122 (2)Å. The formation of a pure phase Na_2_WO_4_ has been confirmed. These refinement results are in good agreement with those reported in the literature for Na_2_WO_4_ ceramic [10]. Figure 1 presents a number of counts versus 2*θ*, where 2*θ* is the angle between the incident and reflected X-ray beams and this spectrum corresponds to an Na_2_WO_4_ pellet sample. The figure displays the accustomed peaks seen in this pellet sample. Sharp and wide peaks refer to large grains and small grains, respectively. The Williamson–Hall (W–H) plot [11] has been used to investigate the crystalline size of Na_2_WO_4_ ceramic. Figure 2 presents a W–H plot for selected peaks of the Na_2_WO_4_ sample. Based on this method, the reciprocal of the broadening is specified by $\beta^{*}=\varepsilon d^{*}+t^{*}$, where d* and t* are the reciprocal of *d* and *t*_,_ respectively. As d* = 0, the reciprocal of the intercept with the *y*-axis estimates the crystalline size and the slope is the strain *ε*. The extrapolation of the best straight line passing through the average data gives an intercept at $t^{*}\approx 3.0012\times 10^{-3}$_,_ with a particle size *t *= 33.3 nm. The slope of that line corresponds to a strain $\varepsilon =0.4953\times 10^{-3}$.

### 3.2. Structure studies

**Figure 3.**
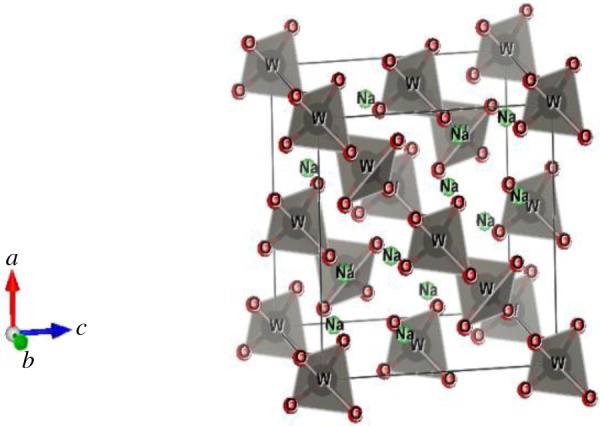
Crystal structure of Na_2_WO_4_ compound in the Fd-3 m structures.

Figure 3 exhibits the crystal structure of Na_2_WO_4_ for easy viewing. The structure of this compound has the normal spinel type with Na^+^ ions on the 16c sites in octahedral coordination and W^+6^ ions on 8b sites in tetrahedral coordination. The $\text{W}{{\text{O}}_{4}}^{-2}$ tetrahedra has perfect *T*_d_ symmetry with W–O bond lengths of 1.7830 (2)  Å. The unit-cell parameters for this compound are in excellent agreement with the literature [12]. However, the Na_2_WO_4_ structure stands apart as being conspicuously inaccurate, giving significantly longer W–O distances, 1.819 (8) Å, and shorter Na–O distances, 2.378 (8) Å, than the ones reported here or in many other simple tungstates [12]. Indeed, the ionic radii of four-coordinated W^+6^ obtained from our analysis of a large range of crystal structures are nearly identical, being 0.42 Å. The values reported here agree very well with the majority of W–O bond lengths in isolated $\text{W}{{\text{O}}_{4}}^{-2}$ tetrahedral oxyanions from a range of alkali metal and alkaline earth compounds tabulated in the literature [12].

### 3.3. Infrared and Raman spectroscopy investigation

**Figure 4.**
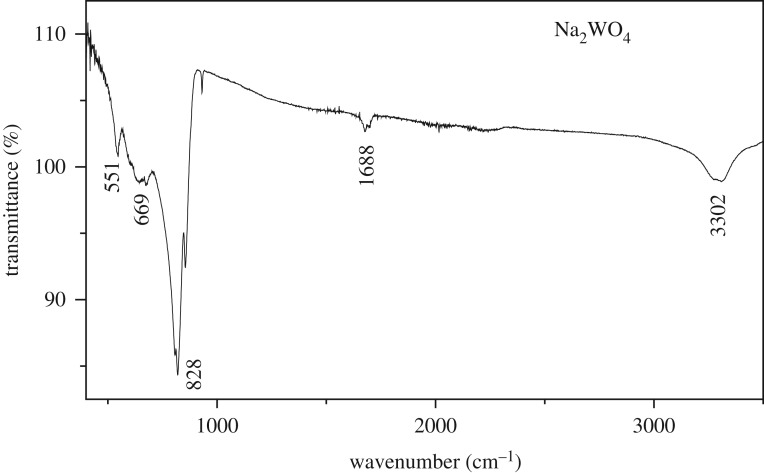
Infrared spectrum of Na_2_WO_4_.

The infrared spectrum of Na_2_WO_4_ is shown in figure 4. Frequencies and assignments of bands are given in tables 1 and 2. The frequencies of the (WO_4_) groups are assigned on the basis of the characteristic vibrations of the O–W–O bridge and $\text{W}{{\text{O}}_{4}}^{-2}$ groups. The assignment of the observed spectral features is based on a comparison with structurally related materials with the following sequence of tungstate vibrations in the order of decreasing frequencies. *υ*_as_ and *υ*_s_ refer to asymmetric and symmetric stretching vibrations of the terminal (WO_4_) or bridging (OWO) bonds, respectively, while *δ* refers to the corresponding bond binding vibrations, which are in general of lower frequency. In these conditions, the peak at 551 cm^−1^ can be attributed to the bending vibration of the $\text{W}{{\text{O}}_{4}}^{-2}$ tetrahedron [13]. The stretching vibration of the $\text{W}{{\text{O}}_{4}}^{-2}$ tetrahedron is observed also in the range 650–800 cm^−1^ [14]. The strong absorption band at 828 cm^−1^ originates from the O–W–O stretches of the WO_4_ tetrahedron. The absorption bands at 1688 and 3302 cm^−1^can be ascribed to the O–H bending and stretching vibrations of the residual water in the samples [15].

**Table 1.** Assignment of infrared bands for Na_2_WO_4_.

wavenumbers (cm^−1^)	attributions
551	*ν*(W_2_O_2_)
669	*ν*(W_2_O_2_)
828	*ν*_as_(W–O)
1688	stretching vibration
3302	stretching vibration

**Table 2.** Assignment of Raman bands for Na_2_WO_4_.

wavenumbers (cm^−1^)	attributions
76	T'L (W_2_O_2_)
317	*δ* (W–O)
375	*δ* (W–O)
818	*ν*_as_ (W–O)
930	*ν*_s_ (W–O)

**Figure 5.**
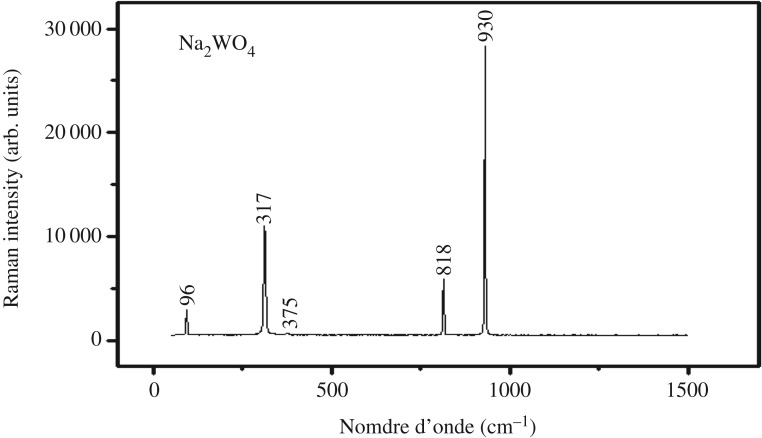
Raman spectrum of Na_2_WO_4_.

The Raman spectrum of the Na_2_WO_4_crystal including all vibrational modes is presented in figure 5. It consists of our strong bands in the 740–1000 cm^−1^ range [16,17], two medium intensity bands in the 270–470 cm^−1^ range (banding vibrations), and a group of medium bands in the 70–260 cm^−1^ range (translational motions of the cations and anions, as well as vibrations of the tungstate polyhedra). The assignment to the respective normal modes is presented in figure 5 [18–22].

The presence of the oxide tungstate groups in the title compound were confirmed by band assignments for the fundamental modes of this compound and are given in table 3.

**Table 3.** The equivalent circuit parameters for the Na_2_WO_4_ sample.

*T* (K)	*R*_gb_10^+5^ (Ω)	*C*_gb_10^−11^ (F)	*R*_g_10^+5^ (Ω)	*Q*_g_10^−11^ (F)	*α*_g_	*R*_el_10^+6^ (Ω)	*Q*_el_10^−10^ (F)	*α*_el_
586	1 0.873	8.04	1.406	2.77	0.966	2.44	2.17	0.863
591	1 0.651	8.58	1.315	3.22	0.953	2.01	2.10	0.871
596	1.352	8.86	1.144	3.45	0.949	1.69	2.11	0.871
601	1.148	9.28	1.029	3.83	0.941	1.40	2.08	0.875
607	0.963	9.01	0.866	3.85	0.942	1.19	2.08	0.876
612	0.809	9.09	0.753	3.98	0.940	1.01	2.07	0. 878
618	0.688	9.19	0.658	4.53	0.931	8.31	2.04	0.881
623	0.591	9.11	0.559	4.81	0.927	6.87	1.99	0.884
634	0.508	9.08	0.483	5.15	0.923	5.72	1.96	0.887
640	0.428	9.04	0.412	5.36	0.921	4.82	1.94	0.889
646	0.343	8.70	0.305	5.64	0.918	3.24	1.89	0.895
653	0.306	9.04	0.278	6.90	0.905	2.67	1.85	0.900
658	0.270	8.62	0.228	6.13	0.914	2.25	1.89	0.899
665	0.244	8.81	0.206	6.87	0.906	1.87	1.83	0.906
672	0.197	9.43	0.179	8.12	0.896	1.56	1.84	0.905
679	0.161	9.80	0.154	8.83	0.892	1.32	1.90	0.902

### 3.4. Impedance properties analysis

**Figure 6.**
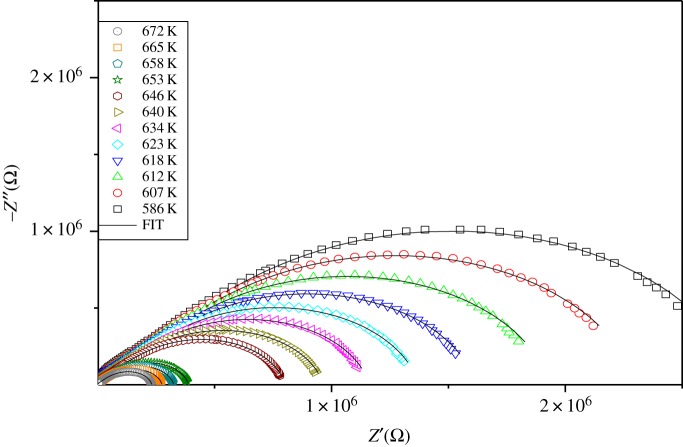
Nyquist plots (−*Z*′′ versus *Z*′) at different temperatures for the Na_2_WO_4_ compound.

The impedance spectroscopy is one of the most important experimental techniques to determine the electrical properties of the sample and to resolve the contributions of various processes such as bulk, grain boundary and electrode effects in the specified frequency domain. This technique is useful to estimate the resistivity and capacitance and it analyses the charge transport processes in the grain–grain boundary of solids. The complex impedance spectra of the Na_2_WO_4_ compound at different temperatures are shown in figure 6. The Nyquist plots of the sample present two semicircles in each impedance spectrum.

This spectrum can be interpreted by means of an equivalent circuit. Ideally, such a semicircle arc passes through the origin of a complex plot and gives a low-frequency intercept on the real axis corresponding to the resistance *R*, of the sample. However, experimental data show a depressed semicircle with the centre below the real axis, which is the reason for using constant-phase elements (CPE) rather than ordinary capacitors in equivalent circuits. The CPE is an empirical impedance function of the type:

$$Z_{\mathrm{CPE}}=\frac{1}{Q{\left(\text{j}\omega \right)}^{\alpha }},$$

where *Q* indicates the value of the capacitance of the CPE element and *α* the degree of deviation with respect to the pure capacitor.

**Figure 7.**
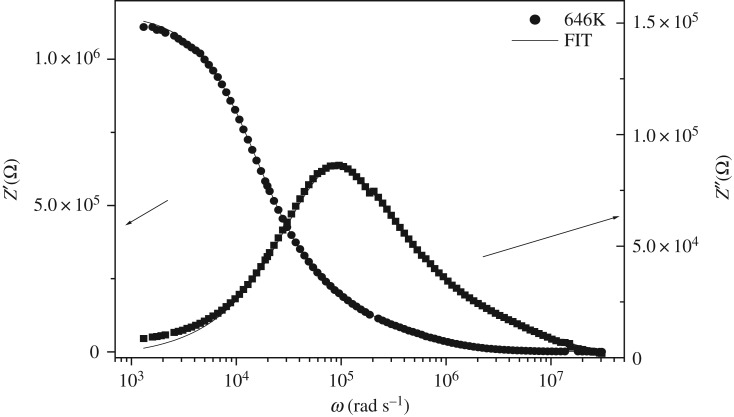
Variation of real and imaginary part of the impedance as a function of frequency and temperature.

The appropriate equivalent circuit to the semicircles consisting of series of a combination of grains and boundary grain elements. Bulk 1 consists of a parallel combination of resistances (*R*_g_) and capacitances (*C*_g_), whereas the grain boundary 1 consists of parallel combination of resistance (*R*_gb_) and constant-phase element CPE_gb_. An appearance of a little semicircle in the low-frequency region can be explained by another combination (*R* parallel CPE). This semicircle is owing to the electrode polarization effect [23]. The equivalent circuit inserted in figure 7 was used to extract the various physical parameters such as the resistance and the capacity of bulk and grain boundaries. Figure 7 shows *Z*′ and *Z*′′ versus the frequencies at different temperatures, which are fitted by equations (3.1) and (3.2). It is observed that the magnitude of *Z*′′ decreases with the increase in temperature and the peak frequency shifts to the higher values. This shift indicates the temperature dependence of the relaxation time. The good conformity of calculated lines with experimental data indicates that the suggested equivalent circuit describes the crystal–electrolyte interface reasonably well:

3.1 $$\begin{array}{rl} Z^{\prime } & \;=\frac{R_{j\text{g}}}{1+{\left(\omega R_{j\text{g}}C_{j\text{g}}\right)}^{2}}+\frac{R_{g}^{2}Q_{g}\omega^{\alpha_{g}}\cos(\alpha_{g}\pi /2)+R_{g}}{{\left(1+R_{g}Q_{g}\omega^{\alpha_{g}}\cos(\alpha_{g}\pi /2)\right)}^{2}+{\left(R_{g}Q_{g}\omega^{\alpha_{g}}\sin(\alpha_{g}\pi /2)\right)}^{2}}\\ & \;\;+\frac{R_{\mathrm{el}}^{2}Q_{\mathrm{el}}\omega^{\alpha_{\mathrm{el}}}\cos(\alpha_{\mathrm{el}}\pi /2)+R_{\mathrm{el}}}{{\left(1+R_{\mathrm{el}}Q_{\mathrm{el}}\omega^{\alpha_{\mathrm{el}}}\cos(\alpha_{\mathrm{el}}\pi /2)\right)}^{2}+{\left(R_{\mathrm{el}}Q_{\mathrm{el}}\omega^{\alpha_{\mathrm{el}}}\sin(\alpha_{\mathrm{el}}\pi /2)\right)}^{2}} \end{array}$$

and

3.2 $$\begin{array}{rl} -Z^{\prime \prime } & \;=R_{j\text{g}}\frac{\omega R_{j\text{g}}C_{j\text{g}}}{1+{\left(\omega R_{j\text{g}}C_{j\text{g}}\right)}^{2}}+\frac{R_{g}^{2}Q_{g}\omega^{\alpha_{g}}\sin(\alpha_{g}\pi /2)}{{\left(1+R_{g}Q_{g}\omega^{\alpha_{g}}\cos(\alpha_{g}\pi /2)\right)}^{2}+{\left(R_{g}Q_{g}\omega^{\alpha_{g}}\sin(\alpha_{g}\pi /2)\right)}^{2}}\\ & \;\;+\frac{R_{\mathrm{el}}^{2}Q_{\mathrm{el}}\omega^{\alpha_{\mathrm{el}}}\sin(\alpha_{\mathrm{el}}\pi /2)}{{\left(1+R_{\mathrm{el}}Q_{\mathrm{el}}\omega^{\alpha_{\mathrm{el}}}\cos(\alpha_{\mathrm{el}}\pi /2)\right)}^{2}+{\left(R_{\mathrm{el}}Q_{\mathrm{el}}\omega^{\alpha_{\mathrm{el}}}\sin(\alpha_{\mathrm{el}}\pi /2)\right)}^{2}}. \end{array}$$

The equivalent circuit modelling gives eight parameters at each temperature (*R*_gb_, *C*_gb_) for grain boundaries, three parameters (*R*_g_, *Q*_g_, *α*_g_) for bulk and (*R*_el_, *Q*_el_, *α*_el_) for electrode effect. The extracted parameters for the circuit elements are summarized in table 3.

#### 3.4.1. Modulus spectroscopy

**Figure 8.**
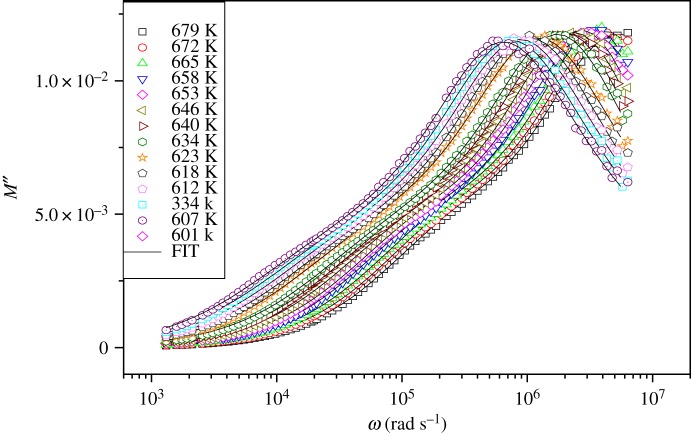
Frequency dependence of the imaginary part of the electric modulus at several temperatures.

Electric modulus formalism is an important theory. It has been used in the analysis of the electrical properties because it gives the main response of the bulk of the crystal sample and is particularly suitable to extract phenomena, and it permits us to study charge transport processes (such as mechanism of electrical transport, conductivity relaxation and ion dynamics as a function of frequencies and temperatures) in ion conductors and eliminates the electrode polarization effect [24]. The electric modulus (M*) is calculated from the following equation:

3.3 $$M^{*}=\frac{1}{\varepsilon^{*}}=\text{j}\omega C_{0}Z^{*}=M^{\prime }+\text{j}M^{\prime \prime },$$

where *M* = *ωC*_0_*Z*, *M*′ = *ωC*_0_*Z*′′ and *M*′′ = *ωC*_0_*Z*′, *C*_0_ is the vacuum capacitance of the cell. The variation of the imaginary part of the modulus (*M*′′) as a function of frequencies at several temperatures is shown in figure 8. It shows two distinct regions which are temperature-dependent. *M*′′ shows a slightly asymmetric peak at each temperature. There are two relaxation peaks located at lower frequencies which are associated with the grain boundary, and at higher frequency with the grain effect. Moreover, when the temperature increases, modulus peak maxima shift to higher frequencies.

Bergman has proposed a function that describes the variation of the imaginary part of the modulus *M*′′ in the frequency domain:

3.4 $$\begin{array}{rl} M^{\prime \prime } & \;=\frac{{M^{\prime \prime }}_{1\max}}{\left((1-\beta_{1})+((\beta_{1})/(1+\beta_{1})))[(\omega_{1\max}/\omega )+({\left(\omega /\omega_{1\max}\right)}^{\beta_{1}})\right]}\\ & \;\;+\frac{{M^{\prime \prime }}_{2\max}}{\left((1-\beta_{2})+((\beta 2)/(1+\beta_{2})))[(\omega_{2\max}/\omega )+({\left(\omega /\omega_{2\max}\right)}^{\beta_{2}})\right]}, \end{array}$$

where $M_{\max}^{\prime \prime }$ and *ω*_max_ are the peak maximum and the peak angular frequency of the imaginary part of the modulus, respectively, and *β* was extracted from the analysis. To account for the grain boundary effects, our experimental data have been modelled with two Bergman functions. The measured data are well fitted to equation (3.4). In the present ceramic sample, *β* may be considered as independent of the temperature in the temperature range studied. For the peaks at low and high frequencies, the *β* parameter values were found to be between 0.18–0.57 and 0.89–0.92, respectively.

## 4. Morphology

**Figure 9.**
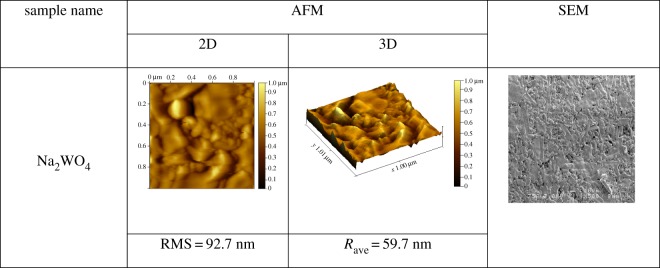
AFM and SEM images of Na_2_WO_4_ sample.

**Figure 10.**
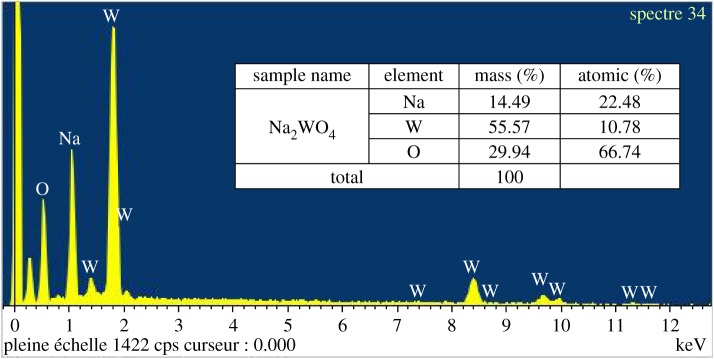
EDX spectra and EDX proportions of Na_2_WO_4_ sample.

**Figure 11.**
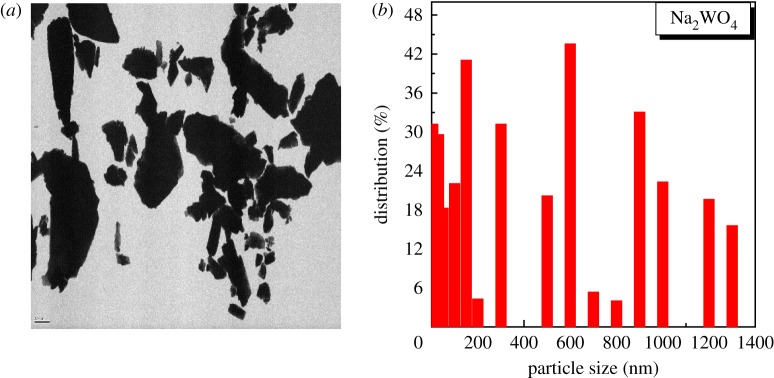
Typical TEM image of the as-synthesized Na_2_WO_4_ sample (*a*) and the corresponding size distribution histogram of the sample particles (*b*) produced in deionized water.

The morphology and microstructure, composition and average particle size of Na_2_WO_4_ sample were investigated by using an atomic force microscope (AFM; Autoprobe CP-RThermomicroscope), scanning electronic microscope (SEM; JSM 6301F, JEOL), energy-dispersive X-ray spectroscopy (EDX; Zeiss EVO LS10) and transmission electron microscopy (TEM; JEM1400, JEOL), respectively, after sintering at 650°C as shown in figures 9–11.

Figure 9 presents two-dimensional (2D) and three-dimensional (3D) AFM images of the Na_2_WO_4_ sample showing a root mean square (RMS) value and an average surface roughness (R_ave_) of about 92.7 and 59.7 nm, respectively.

The SEM image illustrates that Na_2_WO_4_ particles consist of a nanoparticle that creates a homogeneous surface. Figure 10 illustrates EDX data and shows that the atom ratio of Na : W was found to be 2 : 1 which corresponds within the error to the initial chemical composition. This value is in agreement with the refined structure. The compositions of the Na_2_WO_4_ sample was prepared by a solid-state technique using EDX. The figures confirm O, Na and W as the elemental compositions in the inset of figure 10.

Figure 11 shows TEM image and the corresponding size distribution histogram of the Na_2_WO_4_ sample prepared in deionized water. One can see clearly that the prepared sample exhibited a uniform structure, which indicated that Na doping changes the morphologies of Na_2_WO_4_. In addition, the influence of NaCO_3_ on the surface of the tungsten changes the average diameter of the particles of about 600 nm owing to the chemical bounding theory of a single crystal growth.

## 5. Optical properties by means of ellipsometry analysis

Optical experiments provide a good way of examining the properties of semiconductors; especially, the absorption coefficient for various wavelengths gives information about the band gap of the material. Knowledge of these optical gaps is very important for understanding the electrical properties of a semiconductor.

Na_2_WO_4_ samples have been analysed by ellipsometry, which is based on the measurements of the complex ratio *ρ* of the Fresnel reflection coefficients *r*_p_ and *r*_s_ (the reflectances of light parallel and perpendicular to the incident plane (s)), respectively. The standard ellipsometric parameters *ψ* and Δ are given by

5.1 $$\rho =\frac{r_{p}}{r_{s}}=\tan\psi {\text{e}}^{i\Delta }=\tan\psi (\cos\Delta +i\sin\Delta )=f(n,k,t),$$

where Δ = *δ_p_* − δ*_s_* is the phase shift difference, and *f*(*n*, *k*, *t*) an optical function dependent on the layer thickness. The fitting process adopted a new amorphous model, this model is described in the following equations [25,26]:

$$\begin{array}{rl} k(\omega ) & \;=\left\{ \begin{array}{ll} \frac{f_{j}.{\left(\omega -\omega_{g}\right)}^{2}}{{\left(\omega -\omega_{j}\right)}^{2}+\Gamma_{j}^{2}} & \text{for }\omega >\omega_{g},\\ 0 & \text{for }\omega \leq \omega_{g} \end{array}\right.\\ \text{and}\;n(\omega ) & \;=\left\{ \begin{array}{ll} n_{\infty }+\frac{B_{j}.{\left(\omega -\omega_{j}\right)}^{2}+c_{j}}{{\left(\omega -\omega_{j}\right)}^{2}-\Gamma_{j}^{2}} & \text{for }\omega >\omega_{g},\\ 0 & \text{for }\omega \leq \omega_{g}, \end{array}\right. \end{array}$$

where *k*(ω) is the extinction coefficient, *n*(ω) the refractive index, $B_{j}=f_{j}/\Gamma_{j}^{\;}(\Gamma_{j}^{2}-{\left(\omega_{j}-\omega_{g}\right)}^{2})$, *c_j_* = 2.*f_j_*.*Γ_j_*.(*ω* − *ω_g_*), *n*_∞_ the value of the refractive index when (*ω *→ ∞), *f_j_* (eV) the peak of the extinction coefficient, *Γ*_j_(eV) the peak of absorption, *ω_j_* (eV) the energy at which the extinction coefficient is maximum and *ω*_g_ (eV) the optical band gap *E*_g_ of the new amorphous model.

**Figure 12.**
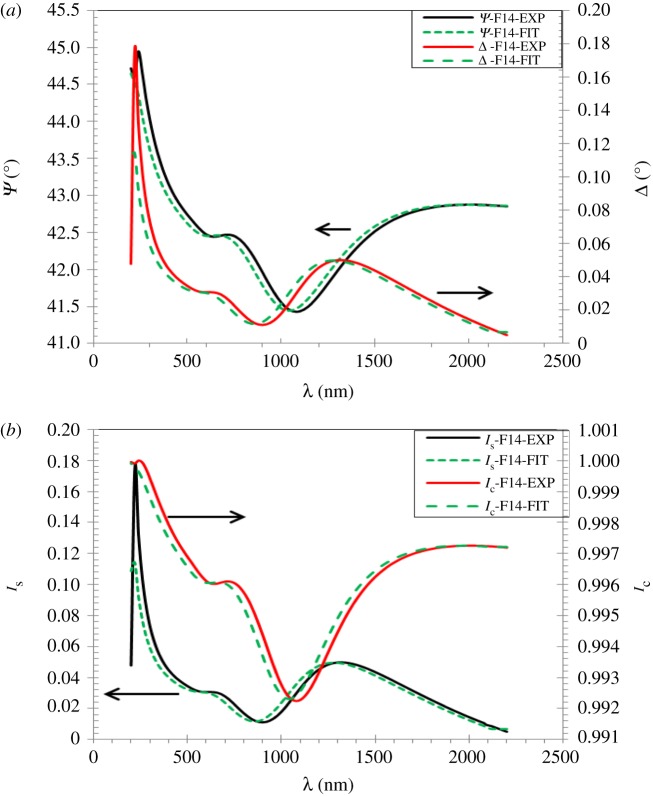
Comparison between the measured and calculated spectra of (*a*) *ψ*, Δ and (*b*) *I*_s_, *I*_c_ as a function of wavelength at angle of incident 70°.

The comparison between the measured and calculated spectra of *ψ*, Δ and *I*_s_, *I*_c_ is shown in figure 12*a*,*b*.

As one can see, the average value of *ψ*_EXP_ and *ψ*_FIT_ in the visible range is about 42.6°–42.5°, respectively, while the average values for Δ_EXP_ and Δ_FIT_ are in the same range of about 0.03° to 0.02°; thus, the standard deviation (*σ*) of the experimental values of *ψ* and Δ gives a fitting value of about 0.07 for *ψ* and 0.007 for Δ values.

In the same method, the average values for $I_{{\text{s}}_{\mathrm{EXP}}}$ and $I_{{\text{s}}_{\mathrm{FIT}}}$ in the visible range are about 0.031–0.028, respectively, while the average values for $I_{{\text{C}}_{\mathrm{EXP}}}$ and $I_{{\text{C}}_{\mathrm{FIT}}}$ are about 0.97–0.99, respectively; thus, the standard deviation for *I*_s_ and *I*_c_ for the experimental values and fitting are about 0.002 and 0.014, respectively. A low *σ* denotes that the data points are inclined to be very close to the mean value (as shown in our case), on the contrary, a high *σ* indicates that the data points are spread out over a large range of values.

The physical parameters for a new amorphous model are presented in table 4, which gives the physical parameters of a new amorphous model; as one can see, the RMS value of *χ*^2^ (MSE) is about 0.81; which mention the differences between the experimental and fitting points for the sample. So, all these experimental and fitted data for the optical parameters of the Na_2_WO_4_ sample are very close according to the value of *χ*^2^ that we acquired. The value of *χ*^2^ should be as small as possible and *χ*^2^ can be defined by

$$\chi^{2}=\min\sum_{1}^{n}\left[\frac{{\left(\Psi_{\mathrm{th}}-\Psi_{\exp}\right)}_{i}^{2}}{\Gamma_{\Psi ,i}}+\frac{{\left(\Delta_{\mathrm{th}}-\Delta_{\exp}\right)}_{i}^{2}}{\Gamma_{\Delta ,i}}\right],$$

where Γ*_i_* is the standard deviation of the points. The smallest value of *χ*^2^ refers to a better fitting results.

**Table 4.** The physical parameters of the new amorphous model for sodium tungstate particles.

model parameters	Na_2_WO_4_
*χ*^2^ (MSE)	0.81
*n*_∞_	7.359 ± 0.063
*ω*_g_	0.261 ± 0.051
*f_j_*	2.229 ± 0.047
*ω_j_*	0.568 ± 0.007
Γ*_j_*	0.001 ± 0.0003
*d* (mm)	0.154 ± 0.214

**Figure 13.**
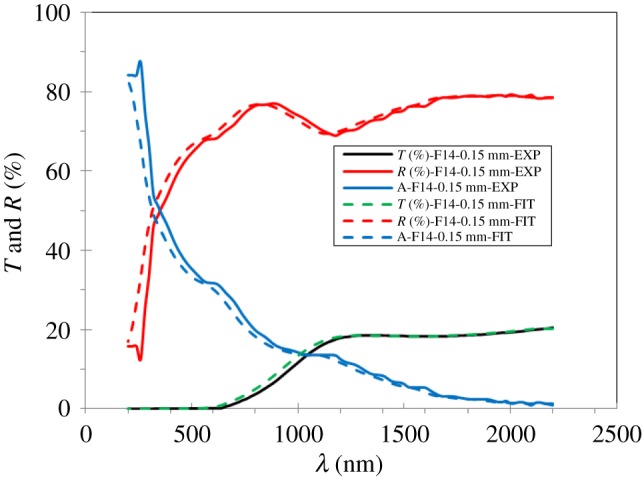
The transmittance, reflectance and absorption spectras for the Na_2_WO_4_ sample measured and calculated.

Figure 13 shows the interpretation of the optical transmittance (*T*), reflectance (*R*) and absorption (*A*) of the Na_2_WO_4_ sample in dependence of the wavelength measured and calculated spectra's.

The average values of the transmittance, reflectance and absorption spectras of the Na_2_WO_4_ sample measured and calculated by SE measurements are in the spectral range of about 200 nm to 2200 nm. These results are obtained with the DeltaPsi2 software.

As one can see, the average measured and fitted transmission value in the visible region for the Na_2_WO_4_ sample are about 0.9% and 1.3% respectively, whereas the average reflectance value is about 68% and 70%, respectively. The average absorptance value is about 31% and 29%, respectively. Thus, a good agreement of the different values for *T*, *R* and *A* of the samples are obtained using ellipsometry (measured and calculated).

Based on the transmission and reflection curves, measurements have been obtained from the new amorphous model, the other optical parameters such as *n*, *k*, *E_g_*, *ε*_r_, *ε*_i_ of the Na_2_WO_4_ sample were investigated experimentally and theoretically.

The absorption coefficient *α* (*λ*) of the Na_2_WO_4_ sample presented according to the optical transmittance curves and the thickness of the sample by the equation

5.2 $$\alpha =\frac{-\ln T}{d}(\text{Lambert}-\text{Beer}),$$

where *T* and *d* are the sample's transmittance and thickness, respectively.

**Figure 14.**
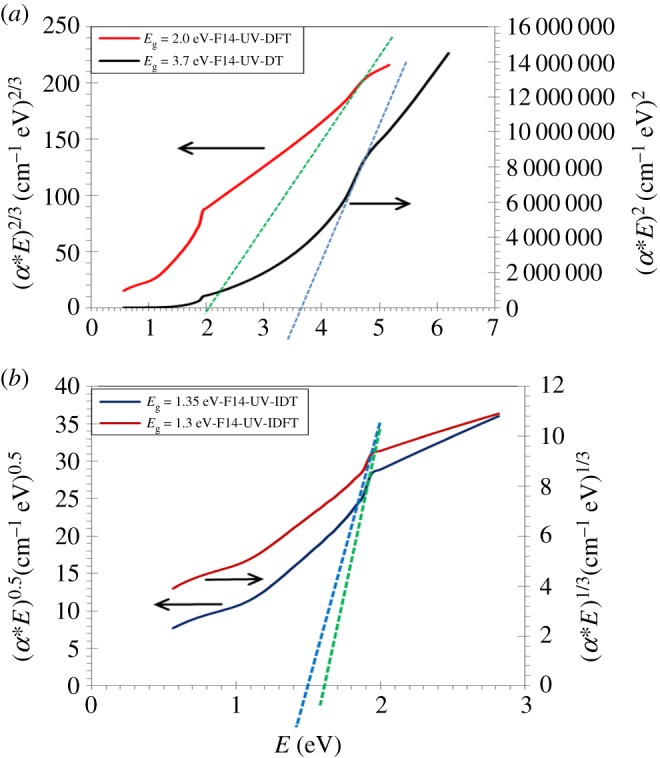
(*a*,*b*) The optical band gap measured of the Na_2_WO_4_ sample for different transition modes. The same procedures are applied to calculate the optical band gap of the sample graphically (theoretically) not shown here.

Therefore, the optical band gap of the Na_2_WO_4_ sample can be determined by Tauc's equation given by the following equation [27]:

5.3 $${\left(\alpha E\right)}^{m}=A(E-E_{g}),$$

where *α* is the absorption coefficient, *A* a constant, *E*_g_ the optical energy gap and *E* the photon energy. We use the absorption coefficient curve as a function of wavelength and the relationship with the incident photon energy. The values *m* = 2, 2/3, 0.5 and 1/3 refer to allowed direct transition (DT), forbidden direct transition (DFT) and allowed indirect transition (IDT) and forbidden indirect transition (IDFT), respectively. By extrapolating the linear part of the plot of versus the values *E* (eV), the optical band gap of the Na_2_WO_4_ sample is determined for *αE* = 0 with this graph. The values of *E*_g_ due to four transition modes of the sample measured and calculated are shown in figure 14*a*,*b* and table 5.

**Table 5.** The optical energy gap of different transition modes measured and calculated.

transition modes	DT		DFT		IDT		IDFT	
sample	*E*_g_EXP__	*E*_g_FIT__	*E*_g_EXP__	*E*_g_FIT__	*E*_g_EXP__	*E*_g_FIT__	*E*_g_EXP__	*E*_g_FIT__
Na_2_WO_4_	3.7	3.7	2.0	2.0	1.35	1.35	1.3	1.3

One can see that an excellent agreement is obtained with the Na_2_WO_4_ sample for the measured and calculated optical energy gap of different transition modes.

The refractive and extinction coefficients *n*(*λ*) and *k*(*λ*) of the Na_2_WO_4_ sample measured and calculated are obtained using the following equations: $n=(1+R(\lambda )/1-R(\lambda ))-\sqrt{4R(\lambda )/{\left(1-R(\lambda )\right)}^{2}-k{\left(\lambda \right)}^{2}}$ and αλ/4π, where *R* is the reflectance, *k* the extinction coefficient, *α* the absorption coefficient and *λ* the wavelength of the light.

**Figure 15.**
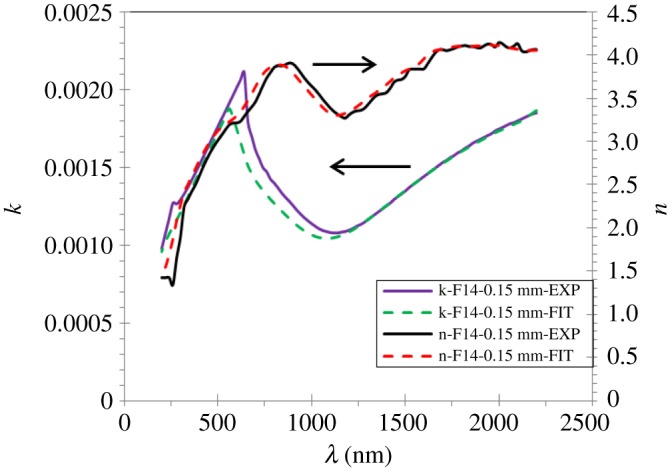
Refraction and extinction indices of the Na_2_WO_4_ sample measured and calculated.

Figure 15 shows the dispersion of the refractive and extinction induced in the dependence of the wavelengths for the Na_2_WO_4_ sample.

One can see the average measured and calculated data of the *n* and *k* in the visible range as a function of the wavelengths of the sample are 3.24, 3.35 and 0.0017, 0.0015, respectively. The refractive index values *n* increase initially with the rises of the wavelength then decrease to the maximum value of 3.5 at 680 nm, and then decrease to the minimum value of 3.3 at 1180 nm and finally increases rapidly with the increase in wavelengths. The extinction coefficient data for the Na_2_WO_4_ sample increase initially to the maximum value of about 0.002 at 640 nm then decrease rapidly towards the shorter wavelength of about 0.01 at 1140 nm, and finally increase with the increase in wavelengths. In addition, *n*_exp_ and *k*_exp_ values for the Na_2_WO_4_ sample obtained by SE are in excellent agreement with those given by FIT points. These results confirmed that a very good model has been used to determine the optical properties of the Na_2_WO_4_ sample.

The complex dielectric constants (real and imaginary) are investigated using the following equations: *ε*_r_ = *n*^2^ − *k*^2^ and *ε*_i_ = 2*nk*.

**Figure 16.**
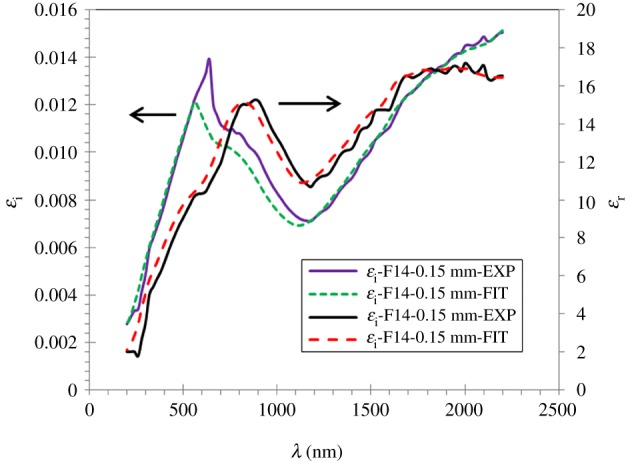
Real and imaginary dielectric constants of the Na_2_WO_4_ sample measured and calculated.

The real and imaginary parts of the dielectric constants of the Na_2_WO_4_ sample are shown in figure 16. This figure showed that *ε*_r_ and *ε*_i_ of the sample decrease with the increase in wavelength.

One can see that the measured and calculated average values of *ε*_r_ and *ε*_i_ in the visible range as a function of the wavelengths are 10.62, 11.34 and 0.01107, 0.01042, respectively. The curves of the measured and calculated real *ε*_r_(*λ*) and imaginary *ε*_i_(*λ*) part of the dielectric constants versus wavelengths of the Na_2_WO_4_ sample are shown in this figure. The average values of *ε*_r_(*λ*) of the sample are in the visible region of about 10.6 and 11.3, whereas the average values of the *ε*_i_(*λ*) are 0.011 and 0.010. The maximum values of $\varepsilon_{{\text{r}}_{\mathrm{EXP}}}(\lambda )$ and $\varepsilon_{{\text{r}}_{\mathrm{FIT}}}(\lambda )$ are 15.3 at 880 nm before decreasing sharply with rises of wavelength. The same behaviour appears with the imaginary dielectric of the sample, the maximum values of $\varepsilon_{{\text{i}}_{\mathrm{EXP}}}(\lambda )$ and $\varepsilon_{{\text{i}}_{\mathrm{FIT}}}(\lambda )$ are 0.014 and 0.01at 640 nm before decreasing sharply with increasing wavelength. Thus, these results revealed a good agreement of the experimental and fitted values of the optical parameters *ε*_r_(*λ*) and *ε*_i_(*λ*) for the Na_2_WO_4_ sample.

**Figure 17.**
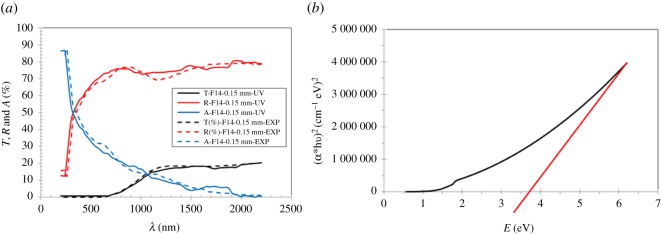
(*a*) The transmittance, reflectance and absorption spectra for the Na_2_WO_4_ sample obtained by spectrophotometry. (*b*) The optical band gap obtained by spectrophotometry.

In addition, the average values of transmittance, reflectance and absorption spectra of the Na_2_WO_4_ sample measured by PerkinElmer Lambda 950 (UV/Vis/NIR) spectrophotometry (integrated sphere) compared with those obtained by ellipsometry (experimental) as shown in figure 17*a*. Based on these curves and equation (5.2), the optical band gap of the sample for indirect transition has been calculated to about 3.7 eV as shown in figure 17*b*, this value is in excellent agreement with that value measured by ellipsometry.

## 6. Conclusion

In the present research work, the Na_2_WO_4_ compound has been synthesized and identified by XRD. The sample was found to crystallize in cubic symmetry with the Fd-3 m space group. The infrared and Raman spectra were recorded and interpreted on the basis of the structural peculiarities of the $\text{W}{{\text{O}}_{4}}^{-2}$.

Characteristically, two semicircles are observed in the impedance plot, indicating the presence of two relaxation processes in the studied compound associated with the grain and grain boundary. Consequently, an equivalent electrical circuit for the electrochemical cell with Na_2_WO_4_ was proposed. The average particle size of about 33.3 nm of the sample has been measured using XRD analysis based on the W–H method. A roughness of about 59.7 nm was found, while SEM images indicate the nanoparticle size of the sample. An EDX study is used to analyse the compositions of that sample and TEM analyses are used to determine the diameter of the particles of about 600 nm.

Finally, the optical properties of the Na_2_WO_4_ particles have been demonstrated using SE measurements and spectrophotometry (integrated sphere) in the spectral range of 200–2200 nm at room temperature. The results that we have obtained from SE (EXP and FIT) showed an excellent agreement. In addition, the optical band gap of Na_2_WO_4_ sample has been calculated by SE and confirmed by spectrophotometry measurements with an excellent agreement too.

## Data accessibility

This article has no additional data.
